# Mast Cell Leukaemia: c-KIT Mutations Are Not Always Positive

**DOI:** 10.1155/2012/517546

**Published:** 2012-09-10

**Authors:** Magalie Joris, Sophie Georgin-Lavialle, Marie-Olivia Chandesris, Ludovic Lhermitte, Jean-François Claisse, Danielle Canioni, Katia Hanssens, Gandhi Damaj, Olivier Hermine, Mohammed Hamidou

**Affiliations:** ^1^Service d'Hématologie, Centre Hospitalier Universitaire, Avenue Laennec, 80054 Amiens, France; ^2^Service de Médecine Interne, Hôpital Européen Georges Pompidou, Université Paris Descartes, Paris Sorbonne Cité, Assistance Publique-Hôpitaux de Paris, 20 Rue Leblanc, 75015, France; ^3^Centre de Référence des Mastocytoses, Faculté de Médecine et AP-HP Necker-Enfants Malades, 156 Rue de Vaugirard, 75743 Paris Cedex 15, France; ^4^Service d'Hématologie Adultes, Université Paris Descartes, Paris Sorbonne Cité, Faculté de Médecine et AP-HP Necker-Enfants Malades, 149 Rue des Sèvres, 75743 Paris Cedex 15, France; ^5^Laboratoire d'Hématologie Biologique et UMR CNRS 8147, Université Paris Descartes, Paris Sorbonne Cité, Faculté de Médecine et AP-HP Necker-Enfants Malades, 161 Rue des Sèvres, 75743 Paris Cedex 15, France; ^6^Laboratoire de Biologie et de Cytologie, Centre Hospitalier Universitaire, Avenue Laennec, 80054 Amiens, France; ^7^Service d'Anatomie-Pathologie, Necker-Enfants Malades, 149 Rue des Sèvres, 75743 Paris Cedex 15, France; ^8^INSERM UMR 891, Centre de Recherche en Cancérologie de Marseille, Laboratoire d'Hématopoïèse Moléculaire et Fonctionnelle, 13009 Marseille, France; ^9^University Hospital of Amiens, Department of Clinical Hematology, Avenue Laennec, 80054 Amiens, Cedex 1, France; ^10^CNRS UMR 8147, Hôpital Necker-Enfants Malades, 149 Rue des Sèvres, 75743 Paris Cedex 15, France; ^11^Service de Médecine Interne, Centre Hospitalier Universitaire, 44000 Nantes, France

## Abstract

Mast cell leukemia (MCL) is a rare and aggressive disease with poor prognosis and short survival time. D816V c-KIT mutation is the most frequent molecular abnormality and plays a crucial role in the pathogenesis and development of the disease. Thus, comprehensive diagnostic investigations and molecular studies should be carefully carried out to facilitate the therapeutic choice. A MCL patient's case with rare phenotypic and genotypic characteristics is described with review of major clinical biological and therapeutic approaches in MCL.

## 1. Introduction

Mastocytosis is a heterogeneous group of disorders characterized by abnormal growth and accumulation of mast cells in one or more organs. The WHO classification [1] placed this entity in the myeloproliferative neoplasms and has the advantage to be clear and serve as a basis to standardize the management of patients with mastocytosis. It defines 7 disease-variants such as cutaneous mastocytosis (CM), indolent systemic mastocytosis (ISM), systemic mastocytosis (SM) with an associated clonal hematological non-mast cell-lineage disease (AHNMD), aggressive SM (ASM), mast cell leukemia (MCL), mastocytic sarcoma, and extracutaneous mastocytoma. The clinical course ranges from “asymptomatic” with normal life expectancy to highly aggressive with worse prognosis [2].

MCL is a rare subtype of the disease which represents less than 2% of mastocytosis. Clinical manifestations are unspecific and share common features with other aggressive SM such as bone lesions, hepatomegaly, spleen enlargement, ascites and enlarged lymph nodes, and rarely mast cell mediator-related symptoms (MCMRS). Cytopenia is a common feature and the presence of high level of atypical marrow MC infiltration exceeding 20% of all nucleated marrow cells is diagnostic criteria. Typically, peripheral mast cells exceed 10% of all nucleated cells; however, aleukemic variants exist and are characterized by less than 10% in the blood and more than 20% MC in the bone marrow. MC morphology has been recognized as prognostic value. Type I mast cell with spindle shape aspect is more often associated with indolent forms; whereas bi or polylobed nuclei or metachromatic blasts are more usual in MCL and aggressive forms [1, 3]. Phenotypic markers are, at least for some authors, of diagnostic value; atypical MCs are positive for CD25 and CD2 [4]. D816V c-KIT mutation is the most frequent abnormality encountered. The presence of this mutation confers a resistance pattern to tyrosine kinase inhibitors such as imatinib. The prognosis is poor with a median survival time of less than 6 months and few effective therapeutic drugs.

We describe one case of MCL without c-KIT mutation suggesting that all c-KIT exons should be sequenced in this entity. We also propose a literature review on the clinical, biological, phenotypic, and molecular specificities as well as on the choice of the therapeutic approach in MCL.

## 2. Patients and Methods

This study was approved by the ethic committee of Amiens University Hospital and was carried out in accordance with the Helsinki convention. Informed consent was obtained.

### 2.1. Case Report

A 36-year-old woman was referred to the Hematology Department for the treatment of mast cell leukemia on May 16, 2011. She complained from a long lasting history (24 months) of severe flushes without evidence of inflammatory, systemic, allergic, or autoimmune diseases. Two months prior her admission, she began to complain from left pelvic pain, which continuously and rapidly increased and was associated with diffuse bone pain and weight loss. These symptoms were preceded by several weeks' of relief from flushes. Chest and abdominopelvic CT scan showed bilateral pleural effusion, large left pelvic mass with psoas muscle infiltration associated with peritoneal irritation, ascites, hepatomegaly, spleen enlargement (20 cm below costal margin), and enlarged abdominal and pelvic lymph nodes. Histological studies of the pelvic mass found a monomorphic infiltration by atypical cells which were negative for CD45, CD20, CD19, CD3, CD100, and cytokeratine.

Full blood counts showed pancytopenia (WBC: 2.8 × 10^9^/L, neutrophils: 1.3 × 10^9^/L, hemoglobin: 6.5 g/dL, platelets: 70 G/L) with no atypical cells on blood smears. Tryptase level was high (400 ng/mL; normal range: <11.5 ng/mL). She required multiple packed red cell transfusions and high dosage of level II analgesics.

### 2.2. Methods

Blood and BM samples were collected at diagnosis for cytopathological, immunophenotypic, cytogenetic, and molecular studies.

Peripheral blood mononuclear cells (PBMC) were isolated by Ficoll-IPaque and stained using FITC, PE, PerCP or APC-coupled anti-CD34(581), CD25(M-A2S1), CD14(MSE2), CD32(3D3), CD2(S5.2), CD4(RPA-T4), CD8(RPA-T8) and CD117(YB5.B8) or with appropriate control, and analyzed by FACS Calibur (all from Becton Dickinson). Anti-CD117 from DAKO, (Carpinteria) was used for the BM immunochemistry. c-KIT mutation screenings were done on total RNA which was isolated from BM aspirate and cDNA was synthesized as previously described [5].

## 3. Results and Discussion

### 3.1. Case Report (Figure 1)

Bone marrow aspirates showed atypical large cells with polylobed nuclei and loose chromatin, representing more than 30% of nucleated marrow cells and classified as type II MC. Trephine marrow biopsy showed hypercellular marrow and the presence of diffuse infiltration by aggregates of atypical polygonal cells with loose chromatin and round nuclei. These cells were positive for CD117 and tryptase but lack CD2, CD2, and CD30 by immunohistochemistry. There was no evidence of associated atypical myeloid, lymphoid cells, or other hematological neoplasm in the marrow. BM immunophenotyping showed a 30% infiltration by MCs which were positive for CD117 and CD9 and negative for CD2 and CD25. Cytogenetic studies revealed a normal karyotype (46XX) and no Bcr-Abl and FIP1-L1/PDGFR-*α* rearrangement by molecular studies. Sequencing of c-KIT in blood and marrow, as previously described [5], showed no evidence of mutation and it was considered as WT c-KIT. Bone mineral density was normal and standard bone radiographics showed diffuse bone osteocondensation. The diagnosis of aleukemic MCL with WT c-KIT was done.

The patient started on methylprednisolone at the dosage of 1 mg/kg per day on May 17, 2011. All symptoms rapidly faded with improvement of bone pain, complete disappearance of the left pelvic pain and the left pelvic mass documented by CT-scan on May 19. We noticed also a mild decrease of spleen and liver sizes. However, blood counts remained unchanged and the patient continued to require red blood supplementation. Corticosteroids were tapered progressively and the patient was planned on a phase 2 clinical trial of KIT tyrosine kinase inhibitor, midostaurin. Unfortunately, the clinical status deteriorated brutally and the patient expired from multiorgan failure in the intensive care unit on May 23, 2012.

### 3.2. Discussion

MCL is by far an incurable disease with an aggressive behavior. It is a rare subtype which represents less than 2% of systemic mastocytosis. The clinical picture is dominated by MC organ infiltration such as hepato-splenomegaly, lymph nodes enlargement, bone abnormalities, ascites, and weight loss. In contrast to indolent mastocytosis, aggressive forms of mastocytosis usually lack skin lesions, and MCMRS are pretty rare. MCMRS are very unspecific, and patients usually complain from multiple symptoms. To the best of our knowledge, isolated flushes have not been reported prior to the development of overt mastocytosis. Thus, systemic mastocytosis should be a differential diagnosis of undiagnosed and chronic flushes. Careful and close followup should also be applied in this situation.

Bicytopenia is frequently encountered in MCL with anemia and thrombocytopenia. The median hemoglobin level is usually around 9 g/dL and the platelet counts of about 70 G/L. Leukopenia and neutropenia are however less common.

Bone marrow infiltration, by atypical mast cell representing more than 20% of all nucleated cells is the hallmark of the disease. Moreover, aleukemic forms are frequent with less than 10% of MCs in the peripheral blood which correspond to the case presented in this paper. MCL may present as de novo with no previous history of mastocytosis. Nevertheless, some cases aroused a variable period of time after the diagnosis or symptoms which may be suggestive of mastocytosis [6]. Transformation to MCL occurred more often from SM-AHNMD or ASM and is correlated to advanced age, history of weight loss, anemia, thrombocytopenia, hypoalbuminemia, and excess of bone marrow blasts. It has also recently been shown that multilineage hematopoietic involvement by KITD816V as well as an immature bone marrow MC phenotype in the absence of coexisting normal MC in the BM might be associated with risk toward progression to more aggressive disease [7, 8].

Atypical MCs express multiple surface antigens such as CD117/kit, CD11c, CD13, CD29, CD33, CD44, CD45, CD63, CD68, and CD71 and are also positive for CD2, CD22, CD25, and CD54. The role of these antigens is however not yet understood. CD2 and CD25 antigens are important markers and their positivity on the surface of MCs constitute minor criteria for the diagnosis of mast cell disease [4]. 

The pathogenesis and prognostic role of CD2 in ASM and MCL has not been elucidated. It has been suggested that CD2 and CD2-CD58-interactions play a role in the abnormal clustering and aggregation of neoplastic MC in the BM [9]. Nevertheless, its prognostic role remains unknown. Decreased expression of CD2 on MC has been reported, at relapse and in late stages of the disease [10]. Thus there is a certain inverse correlation between the expression of CD2 and the aggressiveness of SM as in our case. In MCL, MCs typically lack CD2 expression [4, 11]. In contrast CD25 expression on MCs increased with disease relapse and progression [10]. Our case is atypical by its lack of expression of CD25 on MCs suggesting the absence of implication of CD25 in the aggressiveness and the prognosis of the disease.

CD30 expression on neoplastic MC has however been shown to be correlated to the type and the aggressiveness of SM. CD30 is more often expressed in aggressive SM and MCL than in indolent SM [12]. Nevertheless, it is noteworthy that CD30 expression was also found in some patients with indolent clinical course and alternatively, few patients with CD30 negative MC showed rapid progression. 

Mutation of c-KIT is also a hallmark of the disease. Adult-type human mastocytosis is characterized by mutations in c-KIT at codon 816, which cause constitutive activation of KIT kinase. Different classes of activating KIT mutations respond differentially to KIT inhibitors depending on the site and type of mutation.

D816V c-KIT mutation is the most frequent mutation, found in more than 80% of adult patients with SM especially in the aggressive forms with a frequency of more than 95% in MCL patients. Moreover, WT c-KIT and mutations in exon 8–17 has been reported in few cases [13–21]. This information has important clinical and therapeutic implications. In fact, the D816V c-KIT mutation confers resistance to the available tyrosine kinase inhibitors especially imatinib which is effective in others c-KIT mutations and wild type c-KIT mastocytosis.

Therapeutic approaches are limited and consist of reducing the tumour burden and conservation of organ functions. To date, there is no approved standard therapy to treat SM with the exception of imatinib in D816V c-KIT negative patients.

Corticosteroids have been recommended as a short term use in combination with cytotoxic drugs. It could also be beneficial in some situations such as malabsorption, ascites, or anaphylaxis. Besides its role in alleviating MCMRS, corticosteroids decrease tissue mast cell burden by decreasing tissue stem cell factor [22] and this has been obviously demonstrated in our patient's case with a dramatic tumour response and improvement of symptoms. Thus, corticosteroids could be used as an emergency treatment for ASM and MCL. 

Preliminary studies suggested that *Interferon-*α** (IFN) reduces the frequency of histamine related attacks, decrease BM infiltration by MCs, hepatomegaly, skin manifestations, and improve osteoporosis. However, no major responses were obtained and overall survival was not improved [23–25]. Relapses occurred frequently at the end of therapy and side effects were frequent. As a consequence the dropout rate is very high >25%. Its efficacy in MCL patients is very low or inexistent. *2-Chloro-deoxy-adenosine* has a potential value in the treatment of ASM with symptomatic responses and improvement in mast cell variables. Complete responses are very rare [26, 27]. Cladribine has been used in rare cases of MCL with relatively small or no efficacy [21].

It is now well established that *Imatinib* does not have direct effect on the D816V KIT mutation, but it may affect other sporadic mutations [21, 28, 29]. *Dasatinib* demonstrates significant inhibitory activity against WT KIT as well as juxtamembrane domain mutant KIT [30, 31]. This activity has been proven in patients negative for D816V KIT [32]. *Nilotinib* displays also equipotent activity to Imatinib in D816V c-KIT negative patients and WT Kit cell line [33, 34].

*Masatinib* is a protein-tyrosine kinase inhibitor which, in vitro, potently and selectively inhibits the mutated form, in the juxtamembrane region, of the c-KIT receptor and the c-KIT wild-type receptor. It also inhibits the PDGF receptor and the mutated fibroblast growth factor receptor. Masatinib has been shown to be effective in patients with ISM or CM, not bearing the activating point mutations in the phospho transferase domain of c-KIT with an acceptable toxicity. Recently, Georgin-Lavialle et al. reported on a case of MCL with an exon 9 c-KIT mutation that was successfully treated by masatinib. Thus, this drug should be included in the therapeutic arsenal of mastocytosis patients especially D816V negative. An international randomized phase III in patients with CM and ISM independent of the c-KIT mutation status is ongoing. Finally, *PKC412* (midostaurin) is a small molecule inhibitor of multiple type III receptor tyrosine kinases involved in hematopoiesis and leukemia, an inhibitor of all major isoforms of protein kinase C, the tyrosine kinase associated with the vascular endothelial growth factor receptor. It can also exert inhibitory activity on other mutated tyrosine kinases implicated in a variety of diseases, like KIT (systemic mast cell disease, gastrointestinal stromal tumors) PDGFR, or FGFR1. Midostaurin has shown strong inhibitory activity on neoplastic human MCs carrying the D816V c-KIT mutation in preclinical [35] and clinical settings [36]. Preliminary data of an ongoing phase II trial in patients with advanced systemic mastocytosis treated with 100 mg bid. Midostaurin revealed high overall response rates of 73%. An international phase II study in ASM and MCL is ongoing. 

## 4. Conclusion

MCL is a rare and aggressive disease which can occur de novo or secondary to other mast cell disease and which shares other aggressive systemic mastocytosis features. Careful screening of c-KIT mutation may help in the selection of effective therapy.

## Figures and Tables

**Figure 1:**
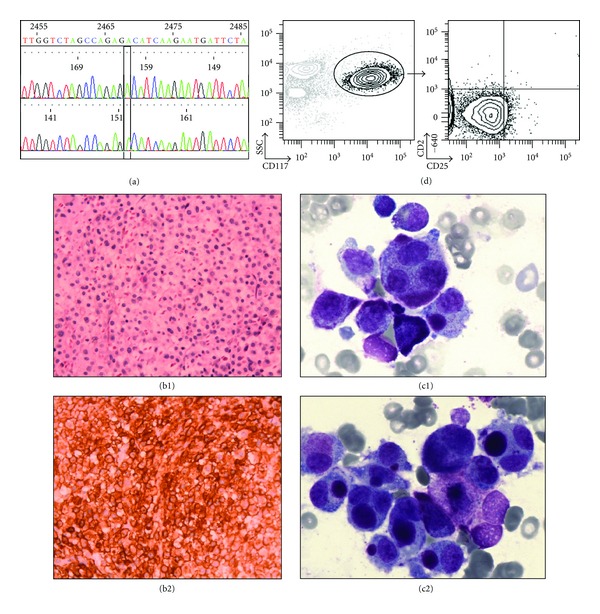
Main features of our patient. (a) Shows the results of a complete c-KIT sequencing: absence c-KIT mutation, especially in exon 17. Indeed, the patient harbored no c-KIT mutation on any exon. (b) shows the results of bone marrow biopsy. (b1): Coloration HES, ×400; (b2) c-KIT immunostaining, ×400. Bone marrow aspirates showed in (c1) a mixture of normal mast cell, leukemic round mast cells, and two very atypical nucleated mast cells with loose chromatin, representing more than 30% of nucleated marrow cell. (c2): cluster of atypical mast cells with a round basophilic mass in the cytoplasm. (d): Immunophenotypic profile of mast-cells characterized by strong KIT/CD117 CD2 and CD25 negative.
